# Haemodynamic effects of prenatal caffeine on the cardiovascular transition in ventilated preterm lambs

**DOI:** 10.1371/journal.pone.0200572

**Published:** 2018-07-11

**Authors:** Corinna Binder-Heschl, Kelly Crossley, Arjan te Pas, Graeme Polglase, Douglas Blank, Valerie Zahra, Alison Moxham, Karyn Rodgers, Stuart Hooper

**Affiliations:** 1 Medical University of Graz, Graz, Austria; 2 The Ritchie Centre, Hudson Institute of Medical Research, Clayton, Victoria, Australia; 3 Department of Pediatrics, Leiden University Medical Centre, Leiden, The Netherlands; 4 Newborn Research Centre, The Royal Women’s Hospital, Melbourne, Victoria, Australia; Hopital Robert Debre, FRANCE

## Abstract

**Background:**

Caffeine is routinely given to preterm infants hours after birth to treat apnea of prematurity. In view of it’s success, earlier administration in the delivery room is being considered, but little is known about how caffeine may effect the cardiovascular changes during the fetal to neonatal transition. Our aim was to determine the effect of prenatal caffeine administration on haemodynamic parameters in ventilated preterm lambs immediately after birth.

**Methods:**

Catheters (carotid artery and jugular vein) and ultrasonic flow probes (pulmonary artery and carotid artery) were implanted in preterm lambs (~126 ±2 days of gestation; term is 147 days), immediately before delivery by caesarean section. Before the cord was clamped, lambs were intubated and a caffeine (10mg/kg caffeine-base; n = 9) or saline (n = 5) infusion was given intravenously to the ewe and lamb over a 15-minute period. Two minutes after clamping the cord, ventilation commenced with a sustained inflation (35 cm H2O for 30 seconds) followed by ventilation for 30 minutes (target tidal volume of 6-8ml/kg).

**Results:**

Blood gas parameters and rectal body temperature were not different between the two groups. Changes in pulmonary blood flow (PBF) and carotid blood flow (CBF) did not differ significantly between groups. PBF increased significantly after ventilation onset in both groups (caffeine p = 0.022, saline p <0.001) and remained elevated thereafter. CBF did not increase but decreased after SI in the caffeine group. Blood pressure, heart rate, and peripheral oxygen saturation did not differ between groups at any stage of the study.

**Conclusion:**

Prenatal caffeine infusion had no significant effect on acute haemodynamic parameters in ventilated preterm lambs during the cardiorespiratory transition.

## Introduction

Caffeine, a methylxanthine, is one of the most widely used medications in neonatology. It acts directly on the respiratory centre to increase respiratory drive and is used as first-line therapy to treat and prevent apnea of prematurity. [1,2] Several studies have shown significant beneficial effects of caffeine in stimulating breathing and reducing apnea in preterm infants. [2–5] Thus, it has been suggested that caffeine administration could occur much earlier after birth, as soon as an intravenous access is obtained, and thereby assist with the cardiorespiratory transition. [6–9]

Caffeine has dose-dependent effects on various biochemical targets in the human body, such as inhibition of phosphodiesterase, blockade of GABA_A_ receptors, mobilization of intracellular calcium and most importantly caffeine acts as a non-specific A_1_ and A_2a_ adenosine receptor antagonist. At therapeutic plasma concentrations, caffeine’s physiological effects are mediated mainly by blocking A_1_ and A_2a_ adenosine receptors. The location of these receptors in the body include the central nervous system, lung, heart and vascular system. [10–12] In adults, caffeine also has haemodynamic effects, including an increase in blood pressure and cardiac contractility. [13,14]

Only a few conflicting studies have investigated the cardiovascular effects of caffeine in preterm infants and none were conducted during transition period. A recent study in preterm infants has demonstrated that caffeine administration within minutes of delivery significantly increases the respiratory effort. [15] This has led to the suggestion that caffeine administration in the delivery room may be beneficial during transition. However, the best route of administration is unclear and the effects of caffeine on the haemodynamic transition are unknown. As caffeine rapidly and freely crosses the placenta, we hypothesize that a caffeine infusion to the mother, prior to delivery, will result in effective fetal plasma caffeine concentrations during transition. [16] Therefore, our aim was to determine the effect of caffeine administration, which commenced prior to delivery, on cardiopulmonary function in ventilated preterm lambs immediately after birth.

## Materials and methods

This study, including all experimental procedures on animals, was approved by Monash University animal ethics committee.

### Surgical preparation

Surgery was performed on 14 anaesthetised pregnant Border-Leicester ewes, bearing single or twin fetuses, at ~126± 2 days of gestation (term is ~147 days). During surgery, fetal lambs were instrumented with catheters and ultrasonic flow probes, immediately before delivery by caesarean section as described previously. [17] Briefly, anesthesia of the ewe was induced by an intravenous injection of 5% sodium thiopentone (Pentothal, 1g/20mL), followed by tracheal intubation and maintenance with inhalation of 1.5%-3% isoflurane in a blended oxygen/air mixture. The ewe was monitored throughout the experiment, recording heart rate, respiratory rate, peripheral oxygen saturation, blood pressure and carbon dioxide levels. The fetal head and neck were exposed via hysterotomy to insert a polyvinyl catheter into the carotid artery (CA) and jugular vein. Ultrasonic flow probes (Transonic Systems, Ithaca, New York, USA) were placed around the non-catheterized CA and left pulmonary artery, with the latter being accessed via a left thoracotomy. [18] Arterial pressure in the CA (CBP) was measured using a pressure transducer (PD10; DTX Plus Transducer; Becton Dickinson, Singapore). All incisions were closed with silk sutures.

A transcutaneous pulse oximeter (Masimo, Irvine, CA) was attached to the right forelimb to measure preductal peripheral oxygen saturation (SpO_2_) levels. The fetal trachea was intubated orally with a 4.0mm cuffed endotracheal tube, which was clamped during surgery to minimize lung liquid loss.

All physiological parameters were recorded continuously (Powerlab; ADInstruments, Castle Hill, NSW, Australia), starting before the caffeine/ saline infusion commenced and continuing until the end of the experiment.

### Experimental procedure

Caffeine base (Auspman, WA, Australia) in a 15mg/mL solution was administered intravenously at 10mg/kg (ewe body weight) over a 15-minute period to the ewe. Just before this infusion finished, an extra 10mg/kg (estimated lamb weight) caffeine infusion was commenced into the lamb via the jugular venous catheter. The control group received the same volume of infusate, which was saline instead of caffeine. Thereafter, the lamb was fully delivered and placed on the ewe’s stomach, taking care to not obstruct catheters or twist flow probes. The endotracheal tube was unclamped and lung liquid was drained passively. Once all physiological recordings had stabilised, the cord was clamped and cut. Thereafter the lambs were closely monitored two-minutes before ventilation commenced, to mimic the time it takes to initiate respiratory support in clinical practice. Ventilation commenced with a 30 seconds sustained inflation (SI) delivered by the Neopuff (Fisher & Paykel Healthcare, Panmure, Auckland, New Zealand) using a peak inspiratory pressure of 35 cmH_2_O and a fraction of inspired oxygen (FiO_2_) of 0.21. After the SI, lambs were connected to the ventilator (Babylog 8000+, Dräger, Lübeck, Germany) and mechanically ventilated for 30 minutes in volume guarantee mode, with a tidal volume of 7mL/kg and a positive end-expiratory pressure of 5 cmH_2_O. The inflation gases were heated and humidified. Ventilation parameters including FiO_2_ were adjusted to maintain SpO_2_ values at 90–95% after the first 10 minutes from birth and to target a PaCO_2_ between 35–55 mmHg, a PaO_2_ between 60–100 mmHg and an arterial pH between 7.30–7.45. Arterial blood gases (0.25mL) were collected before and after caffeine/ saline infusion and 5, 10, 15, 20 and 30 minutes after ventilation onset. To measure caffeine concentrations in plasma, 2.5 mL blood samples were collected before and after caffeine/ saline infusion and at 15 and 30 minutes after ventilation onset and replaced with an equal volume saline.

All lambs were lightly sedated (Alfaxalone i.v. 15mg/kg/h) and remained apneic to allow conduction of the experiment. Antenatal glucocorticoids and postnatal surfactant were not administered to the lambs, as our primarily interest was the cardiovascular change after birth, after receiving prenatal caffeine.

After the experiment the ewes and lambs were humanely euthanized using sodium pentobarbitone (100 mg/kg i.v).

### Data and statistical analysis

Left pulmonary artery blood flow (PBF), carotid artery blood flow (CBF), CBP (mean, systolic and diastolic), SpO_2_ and Heart rate (HR) were averaged over 20 seconds before and after caffeine/ saline infusion, umbilical cord occlusion and applying SI and at 5, 10, 15, 20, 25 and 30 minutes after the ventilation onset.

All physiological recorded data were compared over time and between groups using a two-way ANOVA for multiple comparisons with a Bonferroni post hoc test. An unpaired t test or a Fisher’s exact test was used to compare the descriptive data between the caffeine and saline group. The level of statistical significance was set at p<0.05 and data are presented as mean± SEM.

## Results

Gestational age, birth weight and distribution of males and females did not differ significantly between the caffeine and saline group (Table 1). Similarly, there were no significant differences in blood gas parameters and rectal body temperature between the two groups (Table 2).

**Table 1:** Gestational age, body weight and sex.

	Caffeine group n = 9	Saline group n = 5	p-value
Gestational age (d)	127.3± 0.8	125.2± 0.5	0.077
Male: female ratio	4:5	1:4	0.580
Body weight (kg)	3.33± 0.19	3.21± 0.17	0.685

Data are shown as mean ±SEM.

**Table 2:** Blood gas parameters and body temperature.

	Caffeine group n = 9					Saline group n = 5				
	pH	PaCO_2_	PaO_2_	SaO_2_	Temp.	pH	PaCO_2_	PaO_2_	SaO_2_	Temp.
Before infusion	7.26±0.02	57.4±1.5	22.0±2.2	58.5±5.1	38.8±0.1	7.26±0.02	54.3±4.1	27.5±3.1	68.9±8.2	39.0±0.0
After infusion	7.28±0.01	56.7±1.8	20.7±1.2	57.3±2.2	38.8±0.1	7.28±0.02	53.6±5.8	22.2±2.6	58.3±7.8	38.8±0.2
5min after vent. onset	7.25±0.04	52.4±5.5	41.7±12.3	69.6±11.5	37.5±0.2	7.17±0.04	68.9±8.3	29.7±4.8	66.4±9.3	37.6±0.5
10min after vent. onset	7.29±0.05	46.2±5.2	44.3±7.3	83.2±8.0	37.5±0.3	7.18±0.04	63.8±8.0	45.0±2.7	90.3±1.0	37.2±0.6
15min after vent. onset	7.29±0.04	46.0±4.5	43.3±5.5	89.0±3.2	37.5±0.4	7.21±0.04	61.0±8.0	43.7±1.9	82.9±7.3	37.3±0.7
20min after vent. onset	7.31±0.04	44.0±4.7	45.3±5.1	87.3±4.0	37.3±0.4	7.22±0.04	58.5±8.4	41.6±3.1	89.9±1.4	37.1±0.9
30min after vent. onset	7.32±0.04	45.8±4.2	38.7±4.7	86.3±4.6	37.6±0.4	7.24±0.03	52.9±4.3	40.2±9.4	85.4±4.2	37.2±0.9

Data are shown as mean ± SEM.

### Plasma caffeine concentration

**Fig 1:**
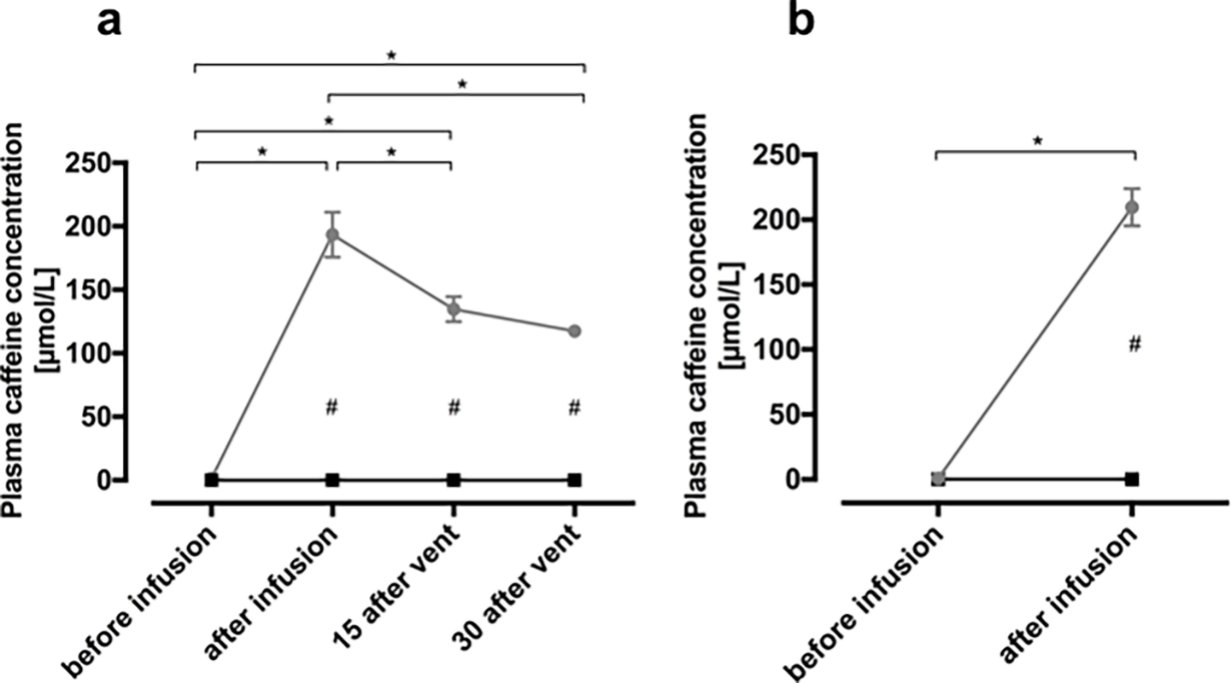
Arterial plasma caffeine concentration in lambs (**a**) and ewes (**b**) before and after caffeine/ saline infusion and at 15 and 30 minutes after ventilation onset (only in lambs). # indicates significant difference between groups. * indicates significant difference within the caffeine group. Data are presented as mean± SEM.

The arterial plasma caffeine concentration was similar between groups before caffeine administration and then increased significantly in ewes and lambs treated with caffeine (Fig 1A and 1B). In caffeine treated lambs, the highest caffeine concentrations were measured immediately following the initial infusion into the ewes and were similar in both ewes and their lambs; in ewes and lambs these values were 209.7 ± 14.5 μmol/L (40.7± 2.8 mg/L) and 193.5 ± 17.6 μmol/L (37.6 ± 3.4 mg/L), respectively. Thereafter, plasma caffeine concentration in lambs decreased to 134.8 ± 9.9 μmol/L (26.2 ± 1.9mg/L) at 15 mins and remained around this concentration for the duration of the ventilation period (30 mins).

### Cardiopulmonary parameters

**Fig 2:**
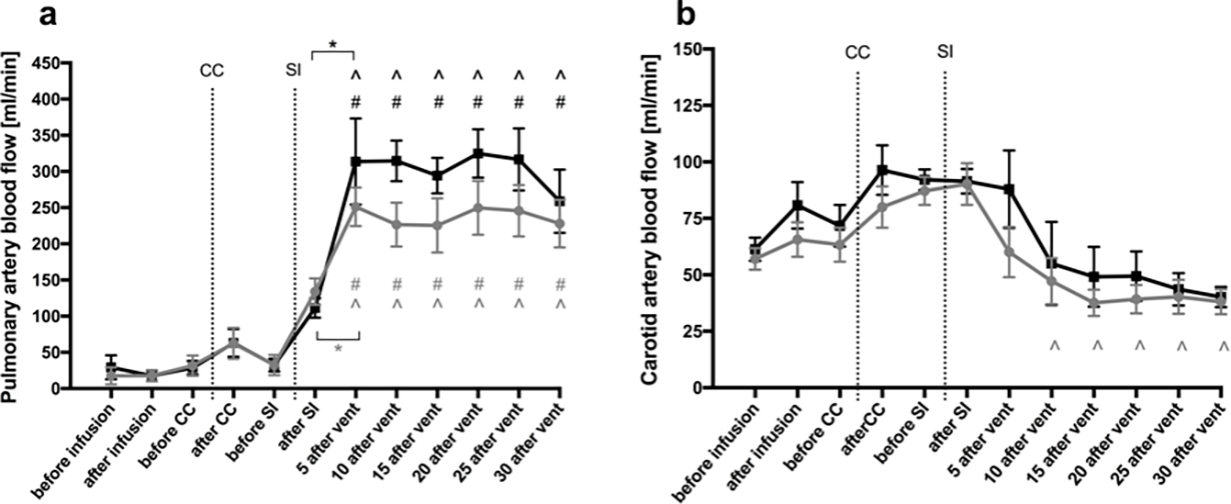
Left pulmonary artery blood flow (**a**) and carotid artery blood flow (**b**) in caffeine treated (grey dots) and control (black squares) lambs. The figure shows mean± SEM values at different time points during the experiment: before caffeine/ saline infusion; after caffeine/ saline infusion; before cord clamping (CC); after CC; before sustained inflation (SI); after SI; 5, 10, 15, 20, 25 and 30 minutes after ventilation onset. * indicates significant difference between two adjacent time points (grey within caffeine group, black within saline group). # indicates significant difference compared to “before CC” (grey within caffeine group, black within saline group). ^ indicates significant difference compared to “before SI” (grey within caffeine group, black within saline group).

In both groups PBF increased significantly after the SI (caffeine p = 0.022, saline p <0.001) and remained elevated for the duration of the experiment (Fig 2A); no differences between groups were observed.

No significant differences in CBF between the groups were observed. CBF tended to increase in both groups following cord clamping, as previously described [19], and remained elevated until after the SI. At this time CBF gradually decreased (Fig 2B).

**Fig 3:**
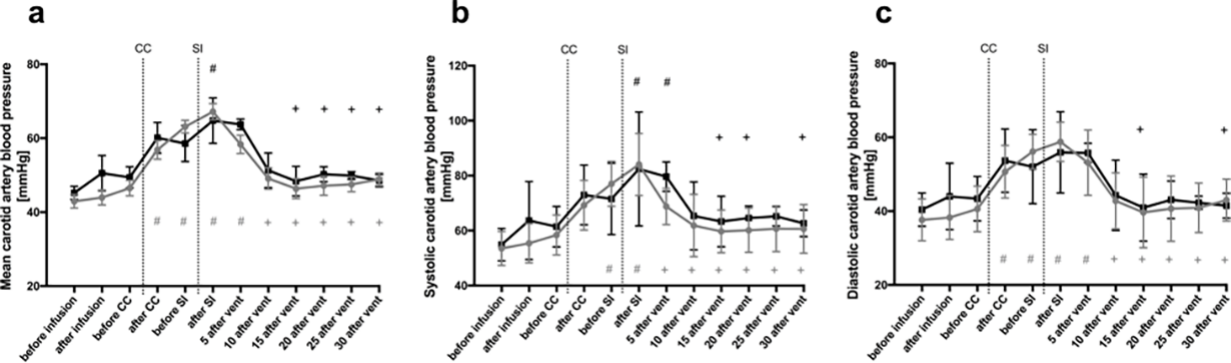
Mean (**a**), systolic (**b**) and diastolic (**c**) carotid artery blood pressure in caffeine treated (grey dots) and control (black squares) lambs. The figure shows mean± SEM values at different time points during the experiment: before caffeine/ saline infusion; after caffeine/ saline infusion; before cord clamping (CC); after CC; before sustained inflation (SI); after SI; 5, 10, 15, 20, 25 and 30 minutes after ventilation onset. # indicates significant difference compared to “before CC” (grey within caffeine group, black within saline group). + indicates significant difference compared to “after SI” (grey within caffeine group, black within saline group).

Mean, systolic and diastolic CBP were not different between saline and caffeine treated lambs (Fig 3A–3C). Cord clamping significantly increased systolic and diastolic CBP, with the highest CBP values achieved shortly after the SI. Thereafter, CBP significantly decrease in both groups. (Fig 3A–3C).

**Fig 4:**
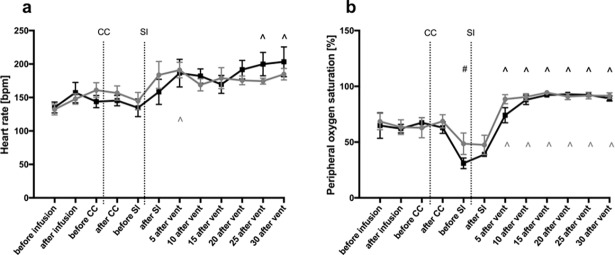
Heart rate (**a**) and peripheral oxygen saturation (**b**) in caffeine treated (grey dots) and control (black squares) lambs. The figure shows mean± SEM values at different time points during the experiment: before caffeine/ saline infusion; after caffeine/ saline infusion; before cord clamping (CC); after CC; before sustained inflation (SI); after SI; 5, 10, 15, 20, 25 and 30 minutes after ventilation onset. # indicates significant difference compared to “before CC” (grey within caffeine group, black within saline group). ^ indicates significant difference compared to “before SI” (grey within caffeine group, black within saline group).

HR was also similar between the groups throughout the study period (Fig 4A). While the HR tended to decrease following CC, the decreases were not significant. However, after receiving the SI, the HR tended to increase in both groups, which was significant in caffeine treated lambs at 5 minutes after ventilation onset. While the increase in HR in saline treated lambs was not significant, the increase closely followed the increase in caffeine treated lambs (Fig 4A).

While the SpO_2_ was not significantly different between groups, the SpO_2_ decreased significantly after CC in the saline, but not in the caffeine treated lambs, reaching a minimum just before the SI. Thereafter, SpO_2_ was significantly increased at 5 minutes of ventilation onset, with the time course for changes in SpO_2_ values being very similar in both caffeine and saline treated lambs (Fig 4B).

## Discussion

Using the fetal sheep model, we wanted to investigate whether a prenatal caffeine infusion effects haemodynamic parameters during the first minutes after cord clamping. Our results showed no significant changes of cardiovascular parameters, such as HR, PBF, CBF, CBP and SpO_2_, in ventilated preterm lambs during neonatal adaption. To our knowledge, this is the first prospective study investigating haemodynamic effects of prenatal caffeine administration immediately after birth.

### Caffeine concentration

In this study we used caffeine-base, administered initially to the ewe and then subsequently to lamb, with the aim of achieving a therapeutic dose during and after the fetal to neonatal transition. The dose initially given to the ewe was 10mg/kg over a 15 min period and was followed by a second infusion of 10mg/kg into the lamb. The first initial dose was based on the ewe’s body weight. As caffeine rapidly and freely crosses the placenta, we expected an almost equal distribution of caffeine concentration between the maternal and fetal compartments, which is essentially what we achieved. Caffeine concentrations in the lamb were similar but slightly below maternal concentrations (40.7± 2.8mg/L vs 37.6± 3.4mg/L) at the end of the initial 15min infusion period. These data indicate that it is possible to preload newborn infants with therapeutic levels of caffeine prior to delivery by administering to the mother. However, as this is dependent upon transplacental caffeine exchange, any factor that disrupts this transfer, such as placental abruption or umbilical cord problems, will reduce the efficacy of this approach.

The total dose administered to the lamb was estimated to be ~20mg/kg of caffeine-base (equivalent to 40mg/kg caffeine-citrate), which is at the upper end of the clinically used dose range. Currently the most widely used loading and maintenance dose of caffeine citrate is 20 mg/kg (equivalent to 10 mg/kg of caffeine-base) and 10 mg/kg (equivalent to 5 mg/kg caffeine-base), respectively. [20] However there are some studies, which have investigated higher doses of caffeine, up to a loading dose of 80mg/kg of caffeine-citrate, to treat apnea of prematurity and to facilitate weaning from mechanical ventilation. [21–23] A study done by Crossley at al investigated slightly higher plasma caffeine concentrations after administration of 40mg/kg caffeine-base directly to the lamb. [24] However, in clinical practice preterm infants reach plasma caffeine concentrations up to 20mg/L and 34mg/L, respectively. [25,26] The critical threshold of caffeine toxicity is >50mg/L and concentrations of 80mg/L are considered lethal. [27]

### Cardiopulmonary and vascular function

We found that all physiological differences examined were similar in saline and caffeine-treated lambs within the first 30 minutes of the transition period. These parameters included mean, systolic and diastolic CBP, PBF, CBF, HR and SpO_2_. To some extent, these findings differ to other studies done in preterm infants and lambs after receiving caffeine. [24,28–30] Crossley et al investigated renal and pulmonary effects after postnatal caffeine infusion during two hours of ventilation in preterm lambs. While they also found no effect on mean arterial pressure, pulmonary artery pressure, PBF and SpO_2_, they did find a significantly higher HR in caffeine-treated lambs, which increased 10 minutes after starting the infusion and remained elevated for the whole experiment. [24] In a study in preterm infants, it was shown that intravenous caffeine administration increased HR, blood pressure and cardiac output. However, this study was not conducted in the immediate newborn period, but rather a week following birth. [30]

A study by Hoecker et al found no meaningful changes in systolic, diastolic and mean blood pressure, HR and left ventricular output in preterm infants one and two hours after caffeine administration. Moreover, they investigated cerebral blood flow velocity and flow velocities in the carotid artery and superior mesenteric artery via Doppler ultrasound one and two hours after caffeine administration and observed a reduction in these parameters. [28] However, most of the human studies exclude infants with an open ductus arteriosus (DA), which may have an influence on cardiopulmonary parameters. In the present study we did not specifically measure DA blood flow, but based on the PBF waveform (mainly due to flows during diastole), we can conclude that the DA was open for the first 30 minutes after birth. Early caffeine administration was associated with a lower incidence of a patent DA in multiple studies. [2,9,31] However, in preterm lambs no direct effect of caffeine on DA contractility could be observed. [32] In our study PBF significantly increased in both groups after SI, but no difference could be observed between the groups. The rapid increase in PBF after ventilation onset results from a very large decrease in pulmonary vascular resistance which redirects the entire right ventricular output into the lungs as well as some left ventricular output via left-to-right shunt through the DA; the later is a direct consequence of the decrease in pulmonary circulatory pressure, which decreases below systemic arterial pressure. [33] Since there was no significant difference in PBF between caffeine and saline treated lambs, a significant effect of caffeine on DA contractility immediately after birth is unlikely. This finding is concordant with a previous study done by Crossley et al. [24]

We observed slightly lower CBF levels in the caffeine group than in the saline group, although the differences weren’t significant. Previous studies reported reduced blood flow velocities in cerebral arteries one to two hours after high-dose caffeine infusions, which were thought to result from vasoconstriction mediated by inhibition of adenosine receptors. [28,34] Cerebral vasoconstriction has also been observed in theophylline treated infants, which was explained by a concomitant decrease in PaCO_2_ [35] although Hoecker et al did not find significant changes of PaCO_2_ values after caffeine treatment. [28] In our study no significant changes in PaCO_2_ were observed before and after caffeine infusion and there were no differences in PaCO_2_ values between the two groups within the study period.

However, we have to admit that the postnatal sedation of the lambs with Alfaxalone differs from typical conditions in humans, though any possible effect on haemodynamics would be equally distributed between both groups.

Caffeine has been shown to stimulate respiratory drive and reduce the number of apneas in preterm infants due to a higher breathing frequency and an improved function of the diaphragm muscle. [1–3] Our experiment was not designed to investigate the effects of caffeine on respiratory function, but rather its effects on cardiovascular parameters during transition. Thus, to avoid the complicating effects of different respiratory function between saline and caffeine treated lambs, we decided to mechanically ventilate to minimize these cofounding effects. A recent clinical trial has clearly demonstrated that caffeine administration in the delivery room significantly improves respiratory function and lung aeration immediately after birth. [15]

### Conclusion

We conclude that a high-dose prenatal caffeine infusion has no acute effect on cardiovascular function immediately after birth in ventilated preterm lambs. Arterial blood pressure and blood flow through the lungs and the brain were similar in both groups, which suggests that there is neither an obvious beneficial nor a harmful effect of caffeine on hemodynamic changes during immediate transition period.
